# Chinese medical entity recognition based on the dual-branch TENER model

**DOI:** 10.1186/s12911-023-02243-y

**Published:** 2023-07-24

**Authors:** Hui Peng, Zhichang Zhang, Dan Liu, Xiaohui Qin

**Affiliations:** College of Computer Science and Engineering, Northwest Normal University, 967 Anning East Road, 730070 Lanzhou, China

**Keywords:** Electronic medical records, Named entity recognition, TENER, Char-Entity-Transformer, Dual-branch

## Abstract

**Background:**

Named Entity Recognition (NER) is a long-standing fundamental problem in various research fields of Natural Language Processing (NLP) and has been practiced in many application scenarios. However, the application results of NER methods in Chinese electronic medical records (EMRs) are not satisfactory, mainly due to the following two problems: (1) Existing methods do not take into account the impact of medical terminology on model recognition performance, resulting in poor model performance. (2) Existing methods do not fully utilize the Chinese language features contained in EMR, resulting in poor model robustness. Therefore, it is imminent to solve these two problems regarding the performance of the NER model for EMRs.

**Methods:**

In this paper, a TENER-based radical feature and entity augmentation model for NER in Chinese EMRs is proposed. The TENER model is first used in the pre-training stage to extract deep semantic information from each layer of the feature extractor. In the decoder part, the recognition of medical entity boundary and entity category are divided into two branch tasks.

**Results:**

We compare the overall performance of the proposed model with existing models on different datasets using the computed F1 score evaluation metric. The experimental results show that our model achieves the best F1 score of 82.67%, 74.37%, 70.16% on the CCKS2019, ERTCMM, and CEMR data sets. Meanwhile, in the CMeEE challenge, our model surpassed the top-3 with the F1 score of 68.39%.

**Conclusions:**

Our proposed model is the first to divide the NER task into a two-branch tasks, entity boundary and types recognition. Firstly, the medical entity dictionary information is integrated into TENER to obtain the feature information of professional terms in Chinese EMRs. Secondly, the features of Chinese radicals in Chinese EMRs extracted by CNN are added to the entity category recognition task. Finally, the effectiveness of the model is validated on four datasets and competitive results are achieved.

## Background

With the rapid development of medical informatization, medical institutions have produced a large number of EMRs texts, which contain critical information about patients in the diagnosis and treatment process, such as chief complaint, diagnosis results, treatment process and drug use. The EMRs contain extremely rich knowledge of clinical experience, which is closely related to the patient’s health status. Effective use of this clinical information can greatly assist physicians in improving the accuracy of their diagnosis. As shown in Table 1, the text information in EMRs is in the form of unstructured data. Among it, the words in bold represent medical entities. Considering this situation, most of the existing methods is to transform unstructured data into structured data. NER is usually used to complete the transformation of unstructured data into structured data, which is the main carrier of relevant medical knowledge in EMRs. NER in electronic medical records is mainly to identify the boundaries of medical entities and determine their categories, which is usually regarded as a task of sequence labeling.

**Table 1:** A labeled example of Chinese EMRs entity recognition

Sample medical record	Entity type
**Outpatient cerebral infarction and subcortical arterioscl-erotic encephalopathy** were admitted to our department.	disease
A small amount of **phlegm sounds** can be heard on bilateral lung auscultation.	symptom
**Head CT** showed high-density foci in the brain parenchyma.	check
**Nasal endoscopic** double sieve, double maxillary sinus.	check
Multiple Myeloma Light Chain Stage **IIIA**	disease diagnosis classification

BERT [1], a pre-trained model based on multilayer Transformer, has achieved great success on several NLP tasks. At present, pre-trained models have received extensive attention from academia and industry as a new paradigm for processing. However, the absolute position encoding, Mask word and FP32 methods used in the training process also affect the performance of BERT in the Chinese medical entity recognition task. Subsequently, TENER (Transformer Encoder for Named Entity Recognition) [2] added position and direction information and introduced relative position encoding based on BERT, which improved the performance of the model in Chinese NER tasks. Therefore, TENER is used as the encoder our proposed model. Considering the particularity of medical data and the important features of medical vocabulary, we integrate the medical entity dictionary information into TENER through the Char-Entity-Transformer method. Through the fusion of word and entity features, the self-attention performance is enhanced to solve the problem of poor pattern recognition performance caused by many ambiguous terms in the medical field.

At the same time, we noticed that some specific types of entity words in the Chinese EMRs data set often have different characteristics from general entity words, especially in the radical kinds of particular entities. Many words that make up the entity words of disease often have the radicals. For example, these words, “scar”, “epilepsy”, “pain”, all have the radical “sickness”. Similarly, many words that make up the entity words of body parts often have the radical “moon”, such as “muscle”, “bone”, “kidney”. Radical information also has specific reference value in predicting labels, especially for medical entities composed of multiple components, such as diseases in the “body part + symptoms” (mouth ulcers) format, which will play a key role. However, the medical NER model has not fully utilized this information. We add radical information as a basic feature to our model to improve the model’s ability to recognize Chinese EMRs.

Compared with traditional text recognition, Chinese EMRs text has many differences. It has the characteristics of concise language and strong structure. In view of these characteristics, this paper proposes a divide-and-conquer solution, which is to identify the boundaries and types of medical entities in Chinese EMRs. Conditional random field (CRF) is used after the TENER encoding layer to identify the boundaries of medical entities. And A-Softmax [3] is used to identify the entity categories. Our contributions can be summarized as follows:

- The proposed model integrates the medical entity dictionary information into TENER for the first time. It solves the problem of poor recognition of medical entities caused by the vague expression of professional terms in EMRs.
- We propose the idea of decomposing the NER task into two-branch tasks of entity boundary and type recognition to solve. In the task of entity category recognition, Chinese radical features are introduced to promote the prediction of entity categories.
- The best F1 score achieved by the experiment was 82.67%, 74.37%, 70.16% on the CCKS2019, ERTCMM, and CEMR data sets. And the proposed model achieved the F1 score of 68.39% in the CMeEE challenge, surpassing the top-3.

## Related work

### Chinese NER

Early NER methods include rule-based methods and dictionary-based methods [4, 5]. With the rise of deep learning, it gradually applied to NER tasks. The method based on deep learning can automatically learn the feature information of the data, so as to obtain the feature representation of the word and complete the entity label prediction. The deep learning based methods are usually divided into dynamic structure and adaptive embedding structure.

Lattice LSTM [6] introduces a compatible LSTM [7] that brings lexical information to Chinese NER tasks. In the LR-CNN [8] model, CNN is used for stacked encoding and rethink mechanism to solve the problem of vocabulary conflict. However, Lattice LSTM and LR-CNN have slow inference speed and cannot capture long-distance dependence. The CGN [9] is developed from the collaborative graph network (GAN). LGN [10] aggregates local and global information to build a graph network, making full use of lexical feature information. FLAT [11] introduces vocabulary information by designing a position vector, using a Transformer to capture long-distance dependence and improve inference efficiency. The above methods belong to dynamic structure methods, but these methods have poor generalization performance.

The adaptive embedding structure is independent of the model and has strong portability. WC-LSTM [12] dynamically encodes the input of Lattice LSTM through four encoding strategies. Multi-digraph [13] introduces entity dictionaries to better model the information interaction between characters and dictionaries through a graph structure. Simple-Lexicon [14] introduces lexical information through the Soft-lexicon method. The model structure is simple and the inference speed is fast.

### TENER

For the NER task, Since Transformer utilizes absolute position encoding, it lacks the ability of direction awareness and does not sufficiently extract contextual feature information. To improve the performance of Transformer on NER tasks, it is critical to improve the position and direction perception characteristics of the Transformer. Inspired by the idea of the relative distance [15] and Transformer-xl [16], a new relative position encoding was used in TENER. It can enhance the model’s ability to sense the direction of contextual feature information. The calculation formula of relative position coding is shown in formula (1).

1 $$\begin{aligned} {} R_t,R_{-t}= \left[ \begin{array}{c} sinc_0t \\ cosc_0t \\ \vdots \\ sin(c_{\dfrac{d}{2}-1}t)\\ cos(c_{\dfrac{d}{2}-1}t)\\ \end{array}\right] , \left[ \begin{array}{c} -sinc_0t \\ cosc_0t \\ \vdots \\ -sin(c_{\dfrac{d}{2}-1}t)\\ cos(c_{\dfrac{d}{2}-1}t)\\ \end{array}\right] \quad \end{aligned}$$

It can be seen from formula (1) that the relative position coding is directional, that is, the relative distance coding in different directions is inconsistent. It is also used in the computation of attention score, as shown in Eq. (2).

2 $$\begin{aligned} {} A^{rel}_{t,j}=Q_tK^T_j+Q_tR^T_{t,j}+uK^T_j+vR^T_{t,j} \end{aligned}$$

where $Q_tK^T_j$ represents the attention score between any two tokens, $Q_tR^T_{t,j}$ represents the offset of the *t*-th token in determining the relative distance, $uK^T_j$ represents the offset of the *j*-th token, and $vR^T_{t,j}$ represents the offset in determining the relative distance and direction.

**Fig. 1:**
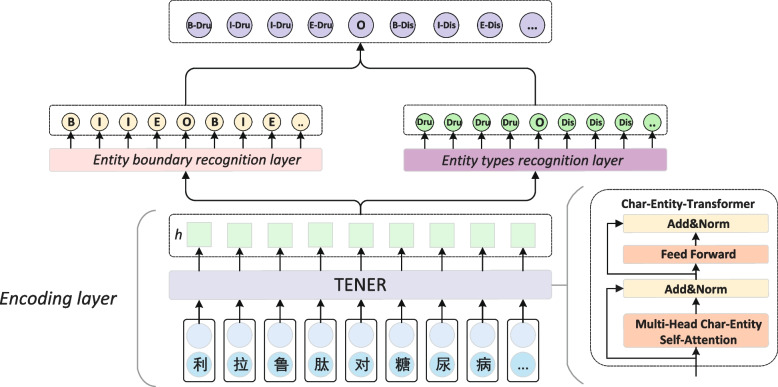
The structure of the dual-branch TENER model. As shown in the Fig. 1, “  ” is the entity’s beginning and “  ” is the end of the entity. Among it, “  ” is labeled as the Dru(Drug), and “  ” is labeled as the Dis(Diseases)

## Methods

The architecture of our proposed model is shown in Fig. 1. In the encoder part, the TENER pre-trained model backbone network is used. The medical entity dictionary information is integrated into TENER to obtain the feature information of professional terms in Chinese electronic medical records. Our model is a two-branch model based on TENER. One of the tasks is the recognition of entity boundary and the other is the recognition of entity types. The boundary information of the entity is obtained through CRF, and the entity category is classified through A-Softmax. The features of Chinese radicals in Chinese EMRs extracted by CNN are added to the entity category recognition task.

### Encoding layer

Given the character sequence $c=\{c_{1},c_{2},...c_{T}\}$ and the entity dictionary $\varepsilon _{ent}$ extracted from the training text, we use the maximum entity matching algorithm to obtain the corresponding entity tag sequence $e=\{e_{1},e_{2},...e_{T}\}$. In particular, we label each character with the index of the longest entity in the $\varepsilon _{ent}$that includes the character, and label characters with no entity matches with 0.

**Fig. 2:**
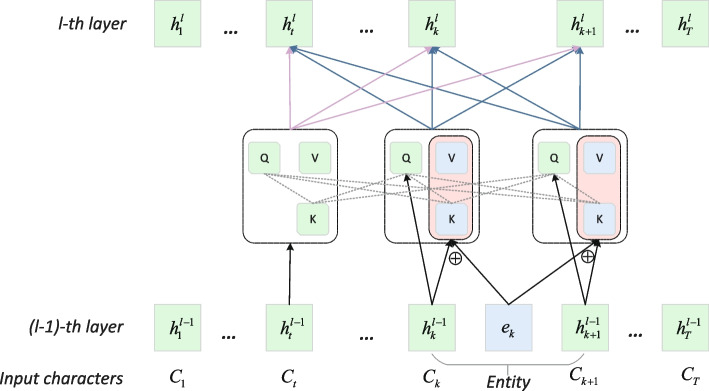
The structure of Char-Entity-Transformer

Figure 2 shows the Char-Entity-Transformer structure. Following TENER, given the character sequence $c =\{c_1,c_2,...c_T\}$, the representation of the *t*-th $(t \in \{1,2,...T\})$ character in the input layer is the sum of character and position embeddings, represented as:

3 $$\begin{aligned} {} h^1_t=E_c[c_t]+E_p[t] \end{aligned}$$

where $E_c$ and $E_p$ represent character encoding table and position encoding table respectively.

Given the $(l-1)$-th layer character hidden sequence $\{h_1^{l-1},h_2^{l-1},...h_T^{l-1}\}$, the calculation process of the *l*-th layer query matrix $Q^l=\{q_t^l\}^T_{t=1}\in \mathbb {R}^{T\times H_c}$ is the same as the query matrix in the traditional self-attention. But for *key* matrix $K^l=\{k^l_t\}_{t=1}^T\in \mathbb {R}^{T\times H_c}$, *value* matrix $V^l=\{v^l_t\}^T_{t=1}\in \mathbb {R}^{T\times H_c}$, we calculate the combination of hidden characters and their corresponding entity coding as follows:

4 $$\begin{aligned} {} q^l_t= & {} h_t^{l-1}W^t_{h,k} \nonumber \\ k_t^l= & {} \left\{ \begin{array}{ll} h_t^{l-1^{T}}W_{h,k}^l &{}\textrm{if } e_t=0,\\ \frac{1}{2} (h_t^{l-1^{T}}W_{h,k}^l+E^T_{ent}[e_t]W^l_{e,k}) &{}\textrm{else};\\ \end{array} \right. \\ v_t^l= & {} \left\{ \begin{array}{ll} h_t^{l-1^{T}}W_{h,v}^l &{}\textrm{if } e_t=0,\\ \frac{1}{2} (h_t^{l-1^{T}}W_{h,v}^l+E^T_{ent}[e_t]W^l_{e,v}) &{}\textrm{else};\\ \end{array} \right. \nonumber \end{aligned}$$

where the $W^t_{h,q}$,$W^l_{h,k}$, $W^l_{h,v} \in \mathbb {R}^{H_c\times H_c}$ are the trainable parameters of the *l*-th layer, and the $W^l_{e,k}$,$W^l_{e,v}\in \mathbb {R}^{H_e\times H_c}$, are trainable parameters for the corresponding entities, and $E_{ent}$ is the entity encoding table.

As shown in formula (4), if a character does not have a corresponding entity, the representation is equal to the baseline self-attention. To illustrate how the character and its corresponding entity are jointly coded, a set of entity codes $\{E_{ent}[e_1],E_{ent}[e_2],...E_{ent}[e_T]\}$ is used, where $e \in \mathbb {R}^{T\times H_e}$. Among it, the attention score $S_i^l$ of the *i*-th character character is as follows:

5 $$\begin{aligned} {} S_i^l= & {} softmax(\frac{q_i^lK^{l^T}}{\sqrt{d_k}}) \nonumber \\= & {} softmax(\frac{q_i^l(h_{l-1}W^l_{h,k}+eW^l_{e,k})^T}{2\sqrt{d_k}}) \nonumber \\= & {} ({\frac{\sqrt{s_t^cs_t^e}}{{\textstyle \sum _{j}^{}\sqrt{s_j^cs_j^e}}}})^T_{t=1} \nonumber \\ s.t. \\ s_t^c= & {} exp(\frac{q_i^l({h_t^{l-1}}^TW^l_{h,k})^T}{\sqrt{d_k}}) \nonumber \\ s_t^e= & {} exp(\frac{q_i^l(e_t^TW^l_{e,k})^T)}{\sqrt{d_k}}\nonumber \end{aligned}$$

where the attention score $s_t^c$ for each character is the same as the traditional self-attention calculation, and the char-to-entity attention score $s_t^e$ represents the similarity between the character and the corresponding entity.

### Entity boundary recognition layer

Generally, in a tag sequence, there are interdependent and mutually constrained relationships between tags. For example, the I (inside) tag should be after the B (begin) tag or the I tag. A set of entity tags should have the same entity type as possible. CRF can describe the dependencies between tags through the transition matrix, and obtain the globally optimal tag sequence.

The optimization goal of the CRF layer is to increase the proportion of the score of the true label sequence to the total. Let $[y]_1^T$ be the true label sequence, and $[j]_1^T$ takes all possible label sequences. The formula for the proportion of the true label sequence to the total is as follows:

6 $$\begin{aligned} {} S([x]_1^T,[i]_1^T)=\sum \limits _{t=1}^{T}(A_{[i]_{t-1},[i]_t}+[M([x]_1^T)]_{{[i]_t},t}) \end{aligned}$$

7 $$\begin{aligned} {} p([y]_1^T|[x]_1^T)=\frac{e^{S([x]_1^T,[y]_1^T)}}{\sum e^{S([x]_1^T,[j]_1^T)}} \end{aligned}$$

8 $$\begin{aligned} {} Loss_C=-S([x]_1^T,[y]_1^T)+log(\sum \limits _{j}^{}e^{S([x]_1^T,[j]_1^T)}) \end{aligned}$$

The loss function is estimated by the negative log-likelihood of the optimization objective, and the calculation formula is as formula (8).

**Fig. 3:**
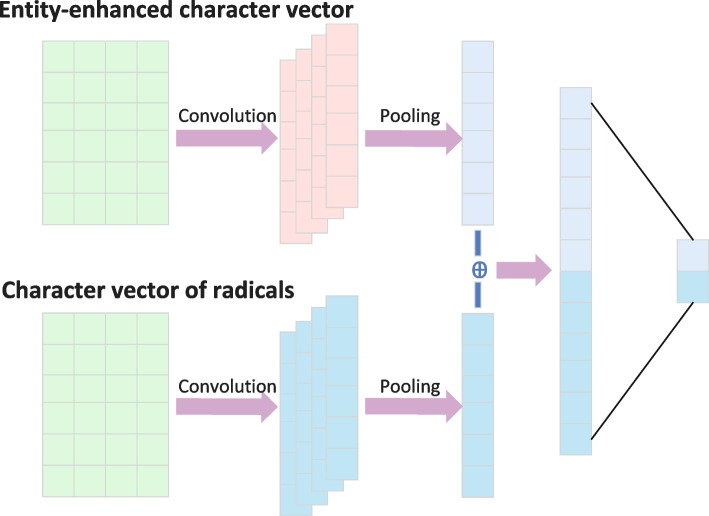
The structure of the radical feature extraction module

### Entity types recognition layer

#### Radical features

Traditional NER models are aimed at generic datasets. The features extracted by the underlying network are limited to the semantic-level features of the context, and lack attention to domain-specific datasets, such as the unique features of Chinese EMRs. Just like in English, you can guess the meaning and nature of the word based on the root and affix of the word. Chinese radicals and strokes also contain a lot of word meaning information, and the flexible use of this information can improve the effect of the model. The difference between Chinese electronic medical record data and general data set lies in the particularity of its domain, so that most of the characters constituting entity words in this type of data are limited and have special characteristics. For example, the Chinese five elements “Gold, Wooden, Water, Fire, Dust” are also often used as the characteristic radicals of various entity words in the medical field. Gold corresponds to the common trace elements in the body and the names of some medicines, such as “$\times \times \times$Na”, “$\times \times \times$ Ca”. Wooden corresponds to “$\times \times \times$surgery”, “physical examination$\times \times \times$”, “$\times \times \times$thrombosis”, “vertebra”, and the names of some Chinese herbal medicines. Water corresponds to various body fluids (plasma, tissue fluid, lymph fluid) and some symptoms, such as “wet”, “slip”, “spill”, “dissolve”, “burst”. Fire is the end character for various inflammation, as well as some symptom entity characters, such as “ scald”, and “rot”. Dust corresponds to “droop”, and body parts modification words “wall”, and so on. In addition, there are others radicals such as “heart”, “car”, “gas” and “mouth”, which also have important reference value for identifying medical entity words.

As shown in Fig. 3, a character-level convolutional neural network (CNN) is designed to capture the fine-grained radical features. It consists of two parts: Entity-enhanced character vector and Character vector of radicals. The CNN structure is the same, and different convolution kernels (2, 3, 4) are selected to extract the key information of the context. The input of the Entity-enhanced character vector part comes from The output of TENER (768 dimensions), the input of Character vector of radicals comes from the Word2Vec (768 dimensions) of the Chinese dictionary. It takes the vector of each character and its radical in the medical data as input, and outputs the sentence vector containing the character and radical information. The obtained character vector is concatenated with the radical vector, and then the concatenated vector is input into the fully connected layer to obtain the result of entity classification. In this way, more and more fine-grained character-level features can be captured.

#### A-Softmax

The Softmax classification algorithm is the most commonly used classification algorithm. The traditional Softmax algorithm uses the same format when learning samples of the same and different classes. This results in poor intra-class and inter-class discrimination of learned features. However, the A-Softmax algorithm increases the difficulty of learning when learning similar samples, which makes the features more distinguishable. This makes predictions for the model difficult and leads to poor classification results. Therefore, we adopt A-Softmax as the classification algorithm for our proposed model. This has a positive impact on the classification of entity types in EMRs. The loss of the entity types recognition layer is as follows:

9 $$\begin{aligned} {}{} & {} Loss_F= \nonumber \\{} & {} \frac{1}{N}\sum \limits _{i}-log(\frac{e^{||x_i||\varphi (\theta _{yi,i})}}{e^{||x_i||\varphi (\theta _{yi,i})}+\sum _{j\ne y_i}^{}e^{||x_i||cos(\theta _{j,i})}}) \end{aligned}$$

Traditional NER tasks in EMRs have only one input and one output. For the two different recognition tasks of entity boundary and type, we use the network structure shown in Fig. 1 to process the two tasks. Finally, the loss of entity boundary and types recognition layer are trained together as the global loss. The specific formula is as follows:

10 $$\begin{aligned} {} Loss=\alpha Loss_B+\beta Loss_F \end{aligned}$$

where the subscripts B and F respectively represent the entity boundary recognition layer and the entity types recognition layer.

### The semi-supervised module

**Fig. 4:**
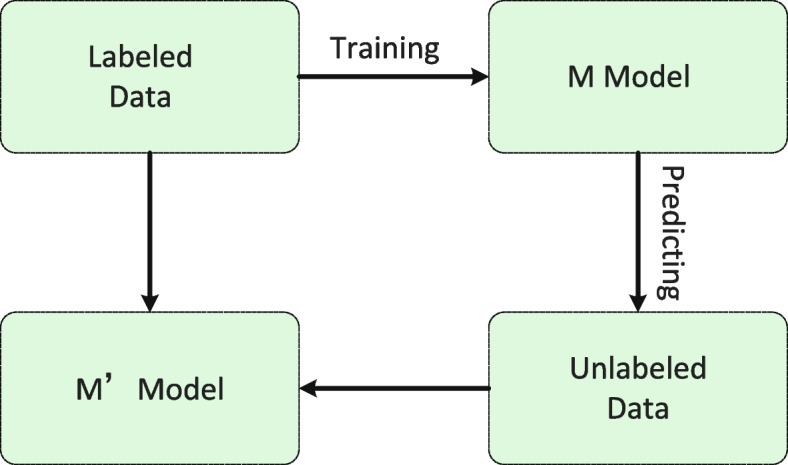
The structure of semi-supervised module

As shown in Fig. 4, the train process of our proposed model mainly includes two steps. The first step is to train the model(M) with a small amount of labeled data. The second step is to use the trained model(M) to predict unlabeled samples. Then the unlabeled samples with high confidence are selected and added to the labeled data iteratively. These two steps are repeated until the preset stopping conditions are reached, and then an ideal model M’ can be obtained. Algorithm 1 is the process of a semi-supervised training method. Parameters of Semi-supervised module shown in Table 2. The loss function calculation process of the semi-supervised training method is shown in Eq. (11).

**Figure:**
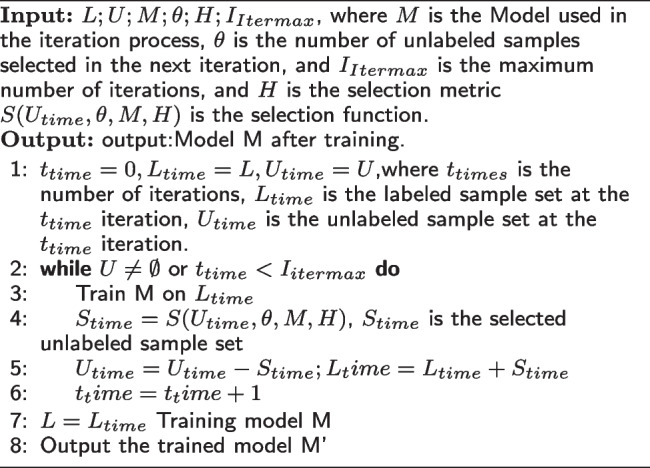
**Algorithm 1** The semi-supervised training process

11 $$\begin{aligned} {} Loss=(1-\theta )\times Loss_{LD} +\theta \times Loss_{ULD} \end{aligned}$$

where the subscripts *LD* and *ULD* respectively represent labeled data and unlabeled data.

**Table 2:** Parameters of semi-supervised module

Parameters	Significations
$L={(x_i,y_i)}$	A label set
$x_i$	The labeled sample, $x_i \in R^d$
$R^d$	The d-dimensional real number set
$y_i$	The true label, $y_i\in \{{w_1,w_2,...w_s}\}$
*s*	The number of different labels
*U*	Unlabeled set
$U=\{{x_1,x_2,...x_m}\}$	*m* is the number of unlabeleds
	amples, $m>n$

## Experiment

We evaluated the proposed model on four datasets, including three publicly available NER datasets (CCKS 2019, ERTCMM, CMeEE) and our own data set (CEMR), which is also one of the contributions of this paper. However, since CEMR contains private data of patients (name, age, home address, etc.), it cannot be made public. The statistics of the four datasets are shown in Table 3.

### Dataset

The total annotated entities in the dataset CCKS2019 are divided on the six types as: Diseases and Diagnosis, Imaging Examinations, Laboratory Tests, Surgery, Drugs, and Anatomical Parts.

The data of ERTCMM (Entity Recognition of Traditional Chinese Medicine’s Manual) comes from the instructions of traditional Chinese medicine, which contains 13 types: Drug, Drug ingredient, Disease, Symptom, Syndrome, Disease group, Food, Food group, Person group, Drug group, Drug dosage, Drug taste, Drug efficacy.

The CMeEE data set is jointly provided by several institutions in China including Peking-University, Zhengzhou University, Pengcheng Laboratory and Harbin Institute of Technology (Shenzhen). It includes 9 types of entity: Disease, Clinical manifestations, Drugs, Medical equipment, Medical procedures, Body, Medical test items, Microorganisms, Departments. The total number of words in the annotated data reaches 2.2 million, which contains 938 files. The average number of words per file is 2355. The data set includes 504 common pediatric diseases, 7085 body parts, 12907 clinical manifestations, and 4354 medical procedures. Slightly different from traditional NER, there is a nested relationship between entities. Nested entities are a common phenomenon in a medical text, so the model processing is more complicated than commonly used NER models.

In order to facilitate the study of medical entity recognition tasks and related topics on Chinese medical record texts, according to the I2B2/VA English medical record text labeling guidelines [17], 4000 real Chinese medical record texts were manually annotated. Finally, it is used as the medical entity recognition dataset (CEMR). All electronic medical records come from the top-3 hospitals in Gansu Province, China. It contains 5 types of entity: Symptoms and characteristics, Examinations, Abnormal examination results, Diseases, and Treatments.

**Table 3:** Statistics of the four datasets

Data Set	Training	Validation	Test	Category
CCKS2019	800	200	379	6
ERTCMM	1200	300	497	13
CMeEE [18]	15000	5000	3000	9
CEMR	3000	500	500	5

### Experimental parameter setup

Each experiment of the model in this paper is repeated three times, and the average result of the ranking is the final result. To be fair, we set the same hyper-parameters (namely, hidden layer size, number of layers, number of attention heads) in the pre-trained model. In addition, unless otherwise specified, for training, all models are trained using the same optimizer Adam.

Adversarial training is used in the model training process, fast gradient method (FGM) and projected gradient descent method (PGD) are used to introduce noise and adjust parameters to alleviate the problem of poor robustness of the model. Finally, the generalization ability of the model is improved. At the same time, mixed-precision training is used to improve the problem of reduced computational efficiency due to adversarial training. FP16 is used for storage and multiplication in the memory to speed up training, and FP32 is used for accumulation to avoid rounding errors. And FP32 is expanded by $2^k$ times before back-propagation loss to prevent the loss from overflowing. During training, the model weights of the last few epochs are weighted averaged to get a smoother and better performing model. To prevent the model from over-fitting, we use 5-fold cross-validation. The differential learning rate is used in the training process. The learning rate of the TENER layer uses 0.00002, and the other layers use 0.02. The parameters of the model are shown in Table 4.

**Table 4:** Hyperparameters

Parameter Name	Value
Clipping gradient	2
The number of TENER encoder layer	24
Dropout	0.3
Hidden size	768
Weight decay	0.01
The number of hidden layer	12
Gradient accumulation steps	2
The number of attention head	12
Hidden activation function	GELU
Epoch	200
Max sequence len	128
Batch size	16

**Table 5:** Overall results of data augmentation, where Rt stands for R-Transformer, TE stands for TENER

	CCKS2019			ERTCMM			CMeEE			CEMR		
Model	P	R	F1	P	R	F1	P	R	F1	P	R	F1
BERT-Rt-CRF	82.42	83.23	82.82	61.42	72.44	66.48	63.45	65.27	64.34	63.04	62.09	62.56
TENER-LSTM-CRF	84.99	87.12	86.02	65.65	74.19	69.66	64.07	67.29	65.64	66.54	64.92	65.72
TENER-Rt-CRF	83.14	81.71	81.20	67.35	75.33	71.12	64.53	63.08	63.79	67.22	66.82	67.03
RoBERTa-TE-CRF	84.11	**87.24**	85.65	67.25	73.22	70.11	62.74	60.11	61.39	66.23	67.06	66.60
RoBERTa-Rt-CRF	85.61	86.24	85.92	67.68	73.45	70.45	62.52	58.02	60.18	67.38	68.65	68.01
ELECTRA-Rt-CRF	**86.18**	86.23	86.20	68.19	76.44	72.08	64.71	63.32	64.00	69.25	66.03	67.60
**Ours**	85.70	87.18	**86.41**	**68.34**	**81.56**	**74.37**	**67.45**	**69.37**	**68.39**	**69.32**	**71.04**	**70.16**

**Table 6:** Experimental results at CCKS2019, where (baseline) is the model comparison baseline

Model	P(%)	R(%)	F1 score(%)
BiLSTM-CRF(baseline)	81.49	80.52	81.00
IDCNN-CRF(baseline)	80.56	81.47	81.01
BERT-CRF(baseline)	81.82	79.33	80.56
Ra-RC(Wu et al.[19])	83.31	82.44	82.87
ELMo-ET-CRF			
(Wan et al.[20])	83.65	**87.61**	85.59
(ELMo)-lattice-LSTM			
-CRF(Li et al.[21])	84.69	85.35	85.02
Ours	**85.70**	87.18	**86.41**

**Table 7:** Experimental results at ERTCMM. where (baseline) is the model comparison baseline, (LSTM) means that the method is based on LSTM

Model	P(%)	R(%)	F1 score(%)
RoBERT-CRF(baseline)	65.79	73.97	69.64
RoBERT-BiLSTM-			
CRF(baseline)	67.56	73.36	70.34
Lattice LSTM	60.52	76.15	67.44
Soft-Lexicon(LSTM)	62.27	75.52	68.26
BERT-Soft-Lexicon(LSTM)	67.01	76.66	71.51
Ours	**68.34**	**81.56**	**74.37**

**Table 8:** Experimental results at CMeEE, the laboratory results are derived from (Zhang et al. [18])

Model	F1 score (%)
BERT-base	62.1
RoBERTa-large[22]	62.1
ALBERT-xxlarge[23]	61.8
ZEN[24]	61.0
MacBERT-base[25]	60.7
MacBERT-large[25]	62.4
PCL-MedBERT[26]	60.6
Human[18]	67.0
Ours	**68.39**

**Table 9:** Experimental results at CEMR

Model	P(%)	R(%)	F1 score(%)
Lattice-LSTM[6]	66.82	70.14	68.44
LR-CNN[8]	65.29	63.84	64.56
CGN[9]	65.92	70.01	67.90
LGN[9]	69.83	66.48	68.11
FLAT[11]	69.26	70.92	70.08
Ours	**69.32**	**71.04**	**70.16**

**Table 10:** The results of the CCKS2019 dataset in the challenge [27]

Model	Disease and diagnosis	An examination	Test	Surgery	Drug	Anatomy	Overall
Top-1	84.29	86.29	76.94	83.33	96.02	86.18	85.62
Top-2	-	-	-	-	-	-	85.59
Top-3	82.81	88.01	75.65	**86.79**	94.49	85.99	85.16
Our	**85.12**	**86.31**	**77.01**	85.42	**96.13**	**86.37**	**86.41**

**Table 11:** The results of the ERTCMM dataset in the challenge [28]

Model	P(%)	R(%)	F1 score(%)
Top-1	67.71	78.95	72.90
Top-2	65.89	81.23	72.76
Top-3	66.60	79.74	72.58
Ours	**68.34**	**81.56**	**74.37**

**Table 12:** The results of the CMeEE dataset in the challenge [29]

Model	F1 score (%)
Top-1	**69.383**
Top-2	69.105
Top-3	68.135
Ours	68.399

**Table 13:** The ablation experiment results on four datasets - F1 score

Model	CCKS2019 F1 score (%)	ERTCMM F1 score (%)	CMeEE F1 score (%)	CEMR F1 score (%)
Ours	**86.41**	**74.37**	**68.39**	**70.16**
- Entity dictionary	82.13	70.26	63.55	67.29
TENER$\longrightarrow$BERT	80.35	64,93	61.04	63.05
Single	85.37	68.32	64.96	68.21
- Radicals feature	83.75	71.02	64.47	66.19
TENER$\longrightarrow$Glove 100d	84.38	66.32	63.96	67.36
A-Softmax$\longrightarrow$Softmax	86.02	69.57	65.75	69.23
- Semi-supervised	86.33	73.41	65.83	70.03

## Results

We summarized the overall performance by computing the F1 score. The results are illustrated in Table 5. We conduct experiments on four datasets using different pre-trained models and downlink encoders. To fairly evaluate the performance of all methods, precision (P), recall (R), and F1 score (F1) are used as evaluation metrics to evaluate the recognition ability of the models.

Table 5 shows the results of the four datasets on the current mainstream pre-trained models. Tables 6 and 10 are the comparisons with the existing state-of-the-art methods and the results of the challenge on the CCKS2019 dataset. Tables 7 and 11 are the comparison results with the existing state-of-the-art methods and the challenge results on the ERTCMM dataset. Tables 8 and 12 are the comparison with the existing methods and challenge results on the CMeEE dataset. Table 9 shows the comparison results between our proposed method and the existing state-of-the-art methods on the CEMR dataset.

In Table 5, although the precision (85.70%) of our model is not the highest, lower than ELECTRA-Rtransformer-CRF (86.18%). This is due to ELECTRA borrowing ideas from GANs and pre-trained reinforcement learning. However, our proposed model achieves the highest F1 score on all four datasets, which demonstrates the superiority of our model over models. In Table 6, the recall of ELMo-TENER-CRF (87.61%) is higher than that of our model (87.18%), which is because there is a small amount of polysemy in the CCKS2019 dataset. Nonetheless, we achieved the highest F1 score. In Table 10, F1 score of the top-3 in “surgery” is 1.35% higher than our model. Our model is 0.79% higher than top-1 (85.62%) overall, and we achieve the highest F1 score on the other three datasets. And our model achieves the best results on the CCKS2019 dataset. In both Tables 7 and 11, our model achieves the highest F1 score. This shows that our model has a good ability to identify multiple medical entity categories (The number of ERTCMM medical entity category is 13, model contains rich external medical entities). The top-1 in the challenge used adversarial training, multi-head selection, pointer network, model fusion and other methods. In Table 8, the F1 score of the human-labeled result is 67.0%, and our model outperforms the human-labeled by 1.39%. In Table 12, we surpassed the top-3 in the challenge (68.135%) by 0.264% and achieved the top-3. This proves that our model can capture long-distance dependence. (The average sentence length in CMeEE dataset is 2355 words). In our annotated dataset CEMR, our proposed model still outperforms other mainstream models. Overall, our model achieves decent experimental results on four different datasets, which demonstrates the superiority of our model.

## Discussion

### Ablation study

To verify the contributions of the three main modules (entity dictionary, dual branch, radical feature), we conduct ablation experiments on four datasets. From the results showed in Table 13, it can be seen that three main modules have improved the performance of this model to varying degrees, where “-” means to delete the component, and “$\longrightarrow$” means to replace the component. “TENER$\longrightarrow$BERT” means to replace the coding layer of TENER with the coding layer of BERT.

**Fig. 5:**
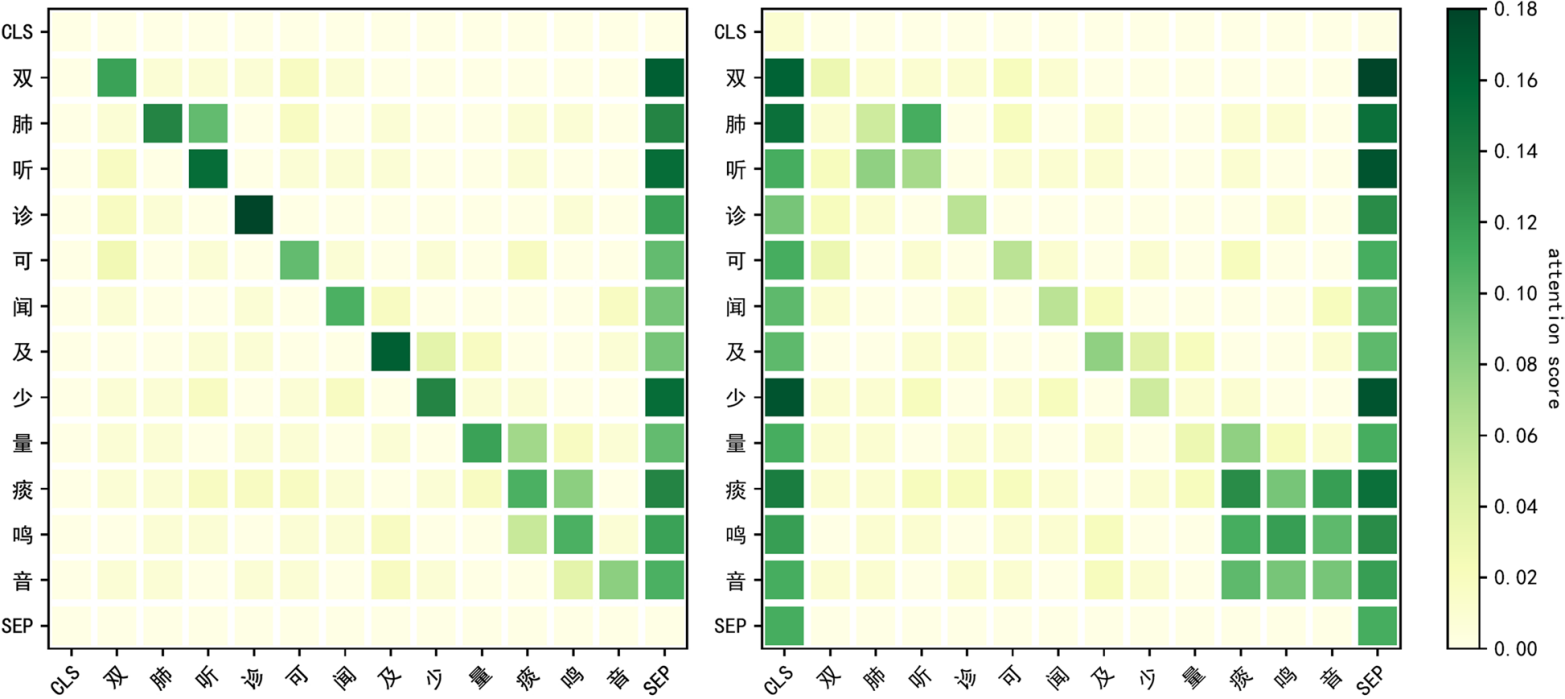
The heat map of attention weight matrix

**Fig. 6:**
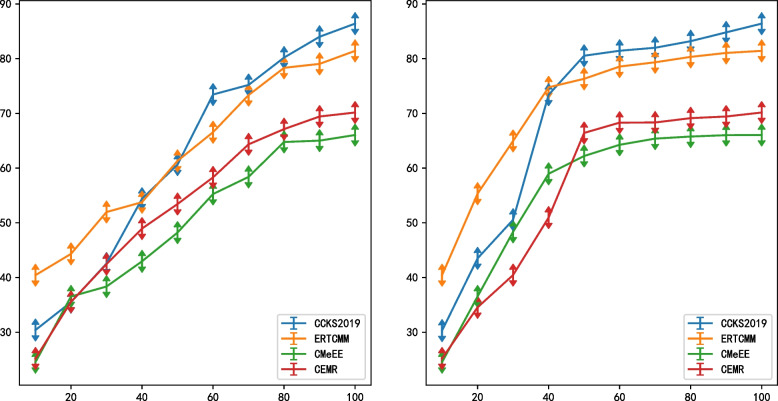
The performance of the model before and after adding the semi-supervised training method

In particular, the addition of our entity dictionary has a specific effect on the model. The entity dictionary collects many medical entities and improves the model’s ability to recognize medical terminology. Since TENER’s whole word masking and relative position coding make the overall performance higher than BERT, the change of character-level encoder has little impact on the result. After merging the dual branches of the model into a single branch, it can be found that the experimental results of the four datasets all drop significantly. This proves that the dual-branch module can effectively identify the boundaries and categories of entities. From the experimental results, it can be found that the experimental results of removing the radical feature module are reduced in all four datasets. This proves that the radical feature can promote the performance of the model to recognize Chinese electronic medical records. The encoding dimension of Glove-100 is much smaller than that of TENER. If the encoded dimension is changed to a larger value, the training speed of the model will decrease significantly. At the same time, we also verified A-Softmax can maintain the compactness within the class and the distance between the classes in the medical entity, and the F1 score on the CEMR data set has increased by 0.93The experimental results show that these three modules can help the model learn Chinese EMRs information. And the semi-supervised model solve the problem of a small amount of data in the clinical field and the particularity of clinical data.

The experimental results show that the three main modules (entity dictionary, dual branch, radical feature) in this model have played an important role. The addition of entity disctionary enables the model to learn more domain knowledge and improve its understanding of context. Dual The branch can better improve the ability of entity recognition and entity classification, and the radial feature module improves the model’s ability to perceive Chinese, which is an important inspiration for the identification of Chinese medical electronic medical records.

Figure 5 is a heat map of the attention weight matrix. The weight matrix used in the heatmap is the attention weights of the 12th multi-head self-attention in the last layer in the TENER module. In the heatmap, the darker the color, the higher the correlation between the two words. Because the weight on the diagonal represents the relationship between each word in the sentence and itself, so the weight is generally larger. Among it, (a) is no the heat map of the weight matrix obtained after the network model of the entity dictionary is coded. (b) is the heat map of the weight matrix obtained after the network model of the entity dictionary is coded. Among them, “phlegm sound” is used as an entity. In (a), the color between each word of the entity is lighter, and the degree of entity association is weak, which leads to the low performance of the model entity recognition. The difference is that in (b) because of The addition of the entity dictionary makes the association between entities close and improves the recognition performance of model entities. When the heat maps are different, it can be clearly found that the proposed model can understand the entity relationship in the sentence at a deeper level. And the entity information enhances the context relationship. This is because the attention mechanism contains entity dictionary information. The pairing of the heat map can be intuitively explained: fusing entity information in the self-attention mechanism can improve the ability of the self-attention mechanism to capture sentence semantics, thereby improving the overall performance of the model.

Figure 6 shows the F1 score of the four data sets. Among them, (a) is the F1 score change of the model with the supervised training method, (b) is the F1 score change of the model with the semi-supervised training method.

We can clearly find that after adding the semi-supervised training method to the model, in the CCKS2019 dataset, the effect achieved by 80% of the data in (a) is similar to the effect achieved by 50% of the data in (b). The same performance is also observed in the other three datasets. Therefore, the use of semi-supervised models can effectively reduce the dependence on the amount of data. Experiments show that the model in this paper has a high recognition ability for medical clinical data when the training data is scarce. It can alleviate model performance degradation due to lack of medical data.

### Case study

We compare the recognition results of the proposed model with three other existing models (BiLSTM-CRF, BERT-CRF, TENER-Rtransformer-CRF) on a case. Table 14 shows a case study on the ERTCMM dataset. Red indicates that the boundary is recognized incorrectly, green indicates that the boundary is recognized correctly, blue indicates that the category is recognized incorrectly, and black indicates that the category is recognized correctly. Among them, “removal of blood stasis” is a “symptom”, “blood stasis” is a “syndrome”, and “hyperplasia of mammary glands” is a “disease grouping”.

**Table 14:** Examples from the ERTCMM test set

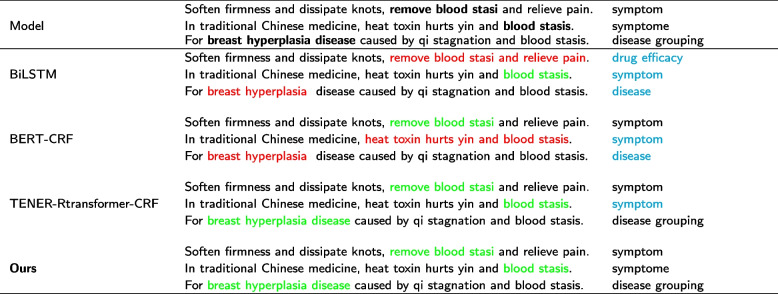	

As can be seen from the Table 14, our model can correctly identify the categories of all three entities, while the other three existing models can only correctly identify a part of the entities. BiLSTM-CRF and BERT-CRF cannot accurately identify entity boundaries. Although TENER-Rtransformer-CRF can accurately identify entity boundaries, it cannot accurately identify entity categories. TENER-Rtransformer-CRF has lower entity recognition performance for similar categories.

## Conclusions

We propose a novel, dual-branch TENER model for entity recognition of Chinese EMRs. Results on the four datasets show that it is highly effective to explicitly integrate the Chinese medical entity dictionary into TENER pre-training. Multiple ablation experiments demonstrate that the dual-branch model formed by adding the entity boundary recognition module is effective for the Chinese medical entity recognition task. And the addition of Chinese radicals makes the model pay more attention to the extraction of unique features of Chinese EMRs. Our method outperforms the state-of-the-art methods for Chinese NER. Worth emphasizing is the limitation of this study is Chinese medical data, and the categories of data entities are distributed evenly. At the same time, there is no Chinese electronic medical record data of nested entities. The existence of polysemy problems (Ambiguity, Lack of context, Out-of-vocabulary words, Overfitting, Data imbalance) has affected the performance of NER, and solving polysemy problems is crucial to improving the accuracy of the NER model. In future work, we can use Context-aware embeddings, Rule-based systems, Named Entity Linking, Domain-specific dictionaries, Ensemble models and other methods to reduce the impact of polysemy on NER. In the future, we aim to increase the processing speed of the model and reduce parameters. Simultaneously, the generalization ability of the model is improved, so that the model has a good performance in multilingual data sets.

## Data Availability

The datasets used and analyzed during the current study are available from the frst author upon reasonable requests. CCKS2019: http://openkg.cn/dataset/yidu-s4k, ERTCMM: https://tianchi.aliyun.com/dataset/86819, CMeEE: https://tianchi.aliyun.com/dataset/95414.

## Abbreviations

BERT: Bidirectional Encoder Representations from Transformers
BiLSTM: Bidirectional long short-term memory
CCKS: China Conference on Knowledge Graph and Semantic Computing
CEMR: Chinese electronic medical record
CGN: Collaborative Graph Network
CMeEE: Chinese Medical Entity Extraction dataset
CNN: Convolutional neural network
CRF: Conditional random field
ELMo: Embeddings from Language Models
EMRs: Electronic medical records
F1: F1 Score
FGM: Fast gradient method
FLAT: Flat-Lattice Transformer
GAN: Generative Adversarial Network
GloVe: Global Vextors
IDCNN: Iterated Dilated convolutional neural network
K: Key
LD: Labeled data
LGN: Lexicon-Based Graph Neural Network
LR-CNN: CNN-Based Chinese NER with Lexicon Rethinking
LSTM: Long short-term memory
NER: Named Entity Recognition
NLP: Natural Language Processing
P: Precision
Q: Query
R: Recall
RoBERT: Robustly Optimized BERT
TENER: Transformer Encoder for Named Entity Recognition
ULD: Unlabeled data
V: Value
WC-LSTM: Word-Character long short-term memory
ZEN: Chinese (Z) text encoder Enhanced by N-gram representations
